# Letter to the Editor: Diagnostic performance of the clear cell likelihood score integrated with cystic degeneration or necrosis on MR imaging for identifying clear cell renal cell carcinoma in cT1 solid renal masses

**DOI:** 10.1186/s13244-026-02314-4

**Published:** 2026-05-29

**Authors:** İlhan Hekimsoy

**Affiliations:** Department of Radiology, Ege University Faculty of Medicine, İzmir, Turkey

Dear Editor,

The investigation by Ning et al [1] regarding the clear cell likelihood score integrated with cystic degeneration (cn-ccLS) offers valuable data for cT1 renal mass characterization. While the authors report improved sensitivity, I wish to highlight four methodological and clinical perspectives that may limit the generalizability of these findings.

First, integrating distinct imaging features raises concerns about pathology. The decision to lump “cystic degeneration” with “necrosis” appears driven by statistics rather than tissue characterization. Since the interobserver agreement for necrosis was weak (kappa = 0.49) [1], combining it with cystic change improves reproducibility but sacrifices accuracy. Necrosis specifically reflects tumor hypoxia and aggression [2], whereas cystic degeneration often represents non-specific fluid accumulation lacking the same prognostic gravity, and treating these disparate features as equivalent results in a pathological oversimplification that creates a misleading risk profile.

Second, the cohort selection introduces a critical diagnostic spectrum bias. The authors conclude that necrosis is significantly associated with clear cell renal cell carcinoma (ccRCC); however, I suggest this association is inflated by the study’s inclusion criteria. By restricting the analysis to only the “five common subtypes,” the study design creates an artificial prevalence that does not reflect real-world clinical practice. In routine scenarios, radiologists encounter a broader spectrum of lesions, including emerging pathological entities recognized by the 2022 World Health Organization (WHO) Classification [3] (e.g., fumarate hydratase [FH]-deficient RCC, renal cell carcinoma not otherwise specified [NOS], and eosinophilic solid and cystic RCC). These specific non-clear cell malignancies characteristically exhibit confounding features (necrosis or cystic changes), yet were omitted from the control group. Consequently, the reported specificity likely reflects the absence of these mimics rather than an intrinsic exclusivity to clear cell histology.

Third, the clinical utility for clinical T1b (cT1b) masses (> 4 cm) appears limited. Although the cn-ccLS improved sensitivity to 98% in this subgroup, current urological guidelines generally favor surgical intervention for solid cT1b masses based solely on oncologic risk, regardless of subtype characterization [4, 5]. Therefore, “upgrading” a cT1b mass to “Likely ccRCC” rarely alters the decision to operate, rendering this statistical improvement clinically neutral.

Finally, the reported predictive values are heavily influenced by prevalence. The positive predictive value (PPV) of 96% is driven by the study’s 78% ccRCC prevalence. If we recalculate the PPV using a more typical clinical prevalence of 60% [6], it drops to 90%. This seemingly minor decrease corresponds to a doubling of the false discovery rate (from 4% to 10%), suggesting the algorithm may yield significantly more false positives in general practice than indicated.

I hope these considerations help readers apply the cn-ccLS to the diverse landscape of renal mass diagnosis.

## Declarations

### Guarantor

The scientific guarantor of this publication is İlhan Hekimsoy.

### Statistics and biometry

No complex statistical methods were necessary for this paper.

### Methodology

- Letter to the Editor
